# Puerarin Attenuates Cadmium-Induced Neuronal Injury via Stimulating Cadmium Excretion, Inhibiting Oxidative Stress and Apoptosis

**DOI:** 10.3390/biom11070978

**Published:** 2021-07-02

**Authors:** Shuangquan Wen, Li Wang, Hui Zou, Jianhong Gu, Ruilong Song, Jianchun Bian, Yan Yuan, Zongping Liu

**Affiliations:** 1 College of Veterinary Medicine, Yangzhou University, Yangzhou 225009, China; 85250@163.com (S.W.); WL18762314639@163.com (L.W.); zouhui@yzu.edu.cn (H.Z.); jhgu@yzu.edu.cn (J.G.); srlbio@163.com (R.S.); jcbian@yzu.edu.cn (J.B.); 2 Jiangsu Co-innovation Center for Prevention and Control of Important Animal Infectious Diseases and Zoonoses, Yangzhou 225009, China; 3 Joint International Research Laboratory of Agriculture and Agri-Product Safety, The Ministry of Education of China, Yangzhou University, Yangzhou 225009, China

**Keywords:** puerarin, cadmium, cadmium excretion, oxidative stress, apoptosis

## Abstract

Cadmium (Cd) is a potential pathogenic factor in the nervous system associated with various neurodegenerative disorders. Puerarin (Pur) is an isoflavone purified from the Chinese medical herb, kudzu root, and exhibits antioxidant and antiapoptotic properties in the brain. In this study, the detailed mechanisms underlying the neuroprotective potential of Pur against Cd-induced neuronal injury was evaluated for the first time in vivo in a rat model and in vitro using primary rat cerebral cortical neurons. The results of the in vivo experiments showed that Pur ameliorated Cd-induced neuronal injury, reduced Cd levels in the cerebral cortices, and stimulated Cd excretion in Cd-treated rats. We also observed that the administration of Pur rescued Cd-induced oxidative stress, and attenuated Cd-induced apoptosis by concomitantly suppressing both the Fas/FasL and mitochondrial pathways in the cerebral cortical neurons of rats both in vivo and in vitro. Our results demonstrate that Pur exerted its neuroprotective effects by stimulating Cd excretion, ameliorating Cd-induced oxidative stress and apoptosis in rat cerebral cortical neurons.

## 1. Introduction

Cadmium (Cd) is an extremely toxic metal pollutant in the environment, which is well known for its occupational health risks. Unlike other metals, Cd is associated with toxic effects at extremely low doses and has a long biological half-life (approximately 30 years in humans) and a low rate of excretion from the body [1]. Growing evidence has shown that Cd exerts its toxic effects on the liver [2], kidney [3], testis [4], bone [5] and brain [6]. Moreover, Cd is a potent neurotoxicant that can penetrate the blood brain barrier (BBB) to enter the brain and neurons [7,8]. Based on published reports and our previous observations, Cd induces neurotoxicity via promoting oxidative stress, apoptosis, and autophagy [6,9,10]. Therefore, it is necessary to identify a novel therapeutic target and strategy to control Cd-induced neuronal injury.

Extensive effort has been made to explore medication for the prevention and treatment of Cd-induced toxicity. Currently, chelation therapy represents the conventional treatment for heavy metal toxicity; however, several safety and efficacy issues remain. Chinese herbal medicine has been effectively employed for millennia for the treatment of various diseases. As science and technology advance, increasing attention has begun to focus on the use of modern separation techniques to extract the active ingredients from Chinese herbal remedies to be used in scientific research [11,12].

Kudzu root (Gegen in Chinese) is the dried root of *Pueraria lobata* (Willd.) Ohwi, which has been used as a traditional Chinese medicine for thousands of years [13]. Puerarin (Pur) is the major bioactive component of the kudzu root, and was first isolated from kudzu root in the late 1950s. Due to its extensive pharmacological activities, Pur has the potential to treat cardiovascular diseases, diabetes, osteoporosis, and liver injury [14,15,16,17]. In addition, Pur can penetrate across the blood–brain barrier and exert various neuroprotective effects [18,19,20]. Previous studies have demonstrated that Pur can alleviate beta-amyloid-induced neurotoxicity through inhibiting PC12 cell apoptosis [21] and protect epilepsy-induced brain injury through antioxidant and antiapoptotic mechanisms [22]. Moreover, isoflavones have been reported to have the potential to alleviate Cd toxicity by stimulating Cd excretion [23,24]. However, whether Pur has a neuroprotective effect on Cd-induced damage remains unknown.

In the present study, we evaluated both the in vivo and in vitro effects of Pur on Cd-induced rat cerebral cortical neuronal damage. We sought to examine whether Pur exerts its neuroprotective effects by stimulating Cd excretion, suppressing Cd-induced oxidative damage and excessive apoptosis.

## 2. Material and Methods

### 2.1. Chemicals and Reagents

Cadmium acetate (229490), poly-L-lysine hydrobromide (P1399), L-glutamine (G8540), and 4′, 6-diamidino-2-phenylindole (DAPI; D9542) were purchased from Sigma-Aldrich (St. Louis, MO, USA). Puerarin (ab142939) was obtained from Abcam (Cambridge, MA, USA). Neurobasal^TM^ medium (21103049), B-27 supplement (17504044), and DMEM/F-12 (12500062) were obtained from Thermo Fisher Scientific (Waltham, MA USA). Trypsin (0458) was obtained from Amresco (Solon, OH, USA). Cell Counting Kit-8 (CCK-8; A311-02) was obtained from Vazyme Biotech (Nanjing, Jiangsu, China). All the antioxidant enzyme detection kits were obtained from the Nanjing Jiancheng Bioengineering Institute (Nanjing, Jiangsu, China). The following primary antibodies were used: Fas (ab82419), Fas Ligand (FasL; ab15285), Fas-associated death domain protein (FADD; ab24533), Cleaved poly (ADP-ribose) polymerase-1 (Cleaved PARP1; ab32064), and apoptosis-inducing factor (AIF; ab1998) were obtained from Abcam (Cambridge, MA, USA). Cleaved Caspase-8 (#9429), Cleaved Caspase-9 (#9507), Cleaved Caspase-3 (#9664), and β-actin (#4970) were obtained from Cell Signaling Technology (Danvers, MA, USA). Bcl-2-interacting domain (BID; NB100-56106SS) was obtained from Novus Biologicals (Littleton, CO USA). All secondary antibodies were obtained from Jackson ImmunoResearch (Philadelphia, PA, USA). Other chemicals and reagents of analytical grade were purchased locally.

### 2.2. Animals and Treatments

A total of 24 five-week-old male Sprague Dawley rats (140 g–150 g) were obtained from the experimental animal center of Jiangsu University (Jiangsu, China). The rats were housed in an environment with well-controlled temperature (23 °C ± 2 °C) and humidity (55% ± 5%), and subjected to a 12-h light and dark cycle. Water and food were provided ad libitum. After acclimatization to these conditions for one week, 24 rats were randomly divided into four groups (six rats/group): (1) control group (given purified water as drinking water; 0.5% carboxymethylcellulose sodium (CMC-Na) administered daily by oral gavage); (2) Pur group (given purified water as drinking water; 200 mg/kg·bw Pur (in 0.5% CMC-Na) administered daily by oral gavage) [18]; (3) Cd group (given purified water containing 50 mg/L Cd [6]; 0.5% CMC-Na administered daily by oral gavage); and (4) Pur and Cd co-treated group (given purified water containing 50 mg/L Cd; 200 mg/kg·bw Pur (in 0.5% CMC-Na) administered daily by oral gavage). In the beginning of the experiment, the rats in the Pur and Pur and Cd co-treated groups were pre-treated with Pur for two weeks, followed by treatment with/without Cd for another 90 days. After 90 days, all of the rats were anesthetized with 2% sodium pentobarbital at 24 h after the last treatment, then sacrificed by cervical decapitation. Blood samples were taken from the aorta ventralis, and the serum was obtained by centrifuging the samples at 2000× *g* for 15 min. The brains were immediately removed and the cerebral cortices were isolated. The tissue was either fixed in 2.5% glutaraldehyde or 4% paraformaldehyde or stored in liquid nitrogen until further analysis. Urine and feces were collected in separators in the metabolic cages during the last week.

### 2.3. Hematoxylin and Eosin (H&E) Staining and Histological Analysis

The brain tissues collected from the rats were fixed in 4% paraformaldehyde at 4 °C for 24 h, and the cerebral cortices were cut into 3 mm-thick sections. The samples were dehydrated in graded solutions of ethanol, immersed in xylol, embedded in paraffin, and sectioned to a thickness of 4 μm. The obtained tissue sections were assembled on glass slides and stained with H&E. All samples were viewed and photographed under a Leica light microscope equipped with a digital camera (DMI3000B, Leica, Germany).

### 2.4. Transmission Electron Microscopy

For the transmission electron microscopy observations, small 1 mm^3^ blocks containing cerebral cortex samples from each group were immediately fixed in ice-cold 2.5% glutaraldehyde overnight at 4 °C. The pieces were rinsed three times in phosphate-buffered saline (PBS) and then post-fixed in 1% osmium tetroxide. Thereafter, the samples were washed three times with PBS, dehydrated in graded solutions of ethanol, and embedded in an epoxy resin. An ultramicrotome (EM UC7, Leica, Germany) was used to obtain ultrathin sections, which were then stained with uranyl acetate and lead citrate. The sections were subsequently examined using a transmission electron microscope (Tecnai 12, Philips, Holland).

### 2.5. Cd Measurement

Rat cerebral cortical tissue was dried, 3 mL of concentrated nitric acid was added, and the tissue was digested in a microwave digestion instrument. The concentration of Cd in each sample was quantified using atomic absorption spectroscopy with inductively coupled plasma mass spectrometry (Optima 7300 DV, PerkinElmer, USA). The Cd concentrations in the serum, urine, and feces were measured using the same method.

### 2.6. TUNEL Staining

Neuronal apoptosis was identified using a terminal deoxynucleotidyl transferase (TdT)-mediated deoxyuridine triphosphate (dUTP) nick-end labeling (TUNEL) assay, in accordance with the manufacturer’s instructions of the In Situ Cell Death Detection Kit^®^ (Roche, Mannheim, Germany). Briefly, paraffin-embedded cerebral cortices were sectioned, followed by deparaffinization in xylene and rehydration in decreasing grades of ethanol. The sections were then incubated with a proteinase K working solution at 37 °C for 25 min, followed by the addition of TUNEL reaction mixture to the samples and an incubation for 2 h at 37 °C in a humidified chamber. After washing with PBS, the samples were incubated with DAB, followed by counterstaining with hematoxylin. Finally, the slides were analyzed by light microscopy equipped with a digital camera (DMI3000B, Leica, Germany).

### 2.7. Cell Isolation and Culture

Fetal Sprague Dawley rats on day 18–19 of gestation were obtained from the experimental animal center of Jiangsu University (Jiangsu, China). Primary rat cerebral cortical neurons were isolated from fetal Sprague Dawley rats on day 18–19 of gestation and cultured as previously described [9,25]. Primary cortical neurons were cultured in neurobasal medium supplemented with 2% B-27 supplement, 1 mM L-glutamine, 100 U/mL penicillin, and 100 μg/mL streptomycin and incubated at 37 °C in 5% CO_2_. Fresh medium was replaced every two days and the cells were used for experiments after six days of culture.

### 2.8. Cell Viability Assay

Primary cortical neurons (3 × 10^4^ cells per well) were seeded into 96-well plates. The cells were pretreated with Pur (100 μM) for 1 h followed by exposure to Cd (10 μM) for 12 h, after which the cell viability was evaluated using a CCK-8 kit according to the manufacturer’s instructions. Cellular absorbance was measured at 450 nm using a Synergy HTX Multi-Mode Microplate Reader (BioTek Instruments, Winooski, VT, USA). 

### 2.9. DAPI Staining

Apoptotic morphological changes in the nuclei of primary cortical neurons were detected by DAPI staining. Primary cortical neurons (5 × 10^5^ cells per well) were seeded onto sterile cover slips placed in six-well plates. After pretreatment with 100 μM Pur for 1 h, the cells were treated with or without 10 μM Cd for another 12 h. Following treatment, the cells were washed with ice-cold PBS, fixed in 4% paraformaldehyde for 20 min at room temperature, and incubated with DAPI staining solution (1 μg/mL in PBS) for 5 min in the dark. After three washes with PBS, the cells were viewed under a fluorescence microscope (TCS SP8 STED, Leica, Germany) to assess the level of chromatin condensation.

### 2.10. Oxidative Stress Assessment

Rat cerebral cortical tissue samples and primary cortical neurons were homogenized and fresh serum samples were prepared. The level of catalase (CAT) activity, glutathione (GSH), and malondialdehyde (MDA) was measured using commercial kits in accordance with the manufacturer’s instructions (Nanjing Jiancheng Bioengineering Institute, Nanjing, China).

### 2.11. Western Blotting

For the tissue samples, the cerebral cortical tissue was homogenized and lysed with protease inhibitors. In the cell samples, primary cortical neurons were treated with 10 μM Cd for 12 h following a pre-incubation with 100 μM Pur for 1 h. The protein samples were extracted by ultrasonication in lysis buffer containing protease inhibitors. An equivalent amount of protein was separated by sodium dodecyl sulfate-polyacrylamide gel electrophoresis (SDS-PAGE) and transferred to a polyvinylidene difluoride (PVDF) membrane. After blocking in 5% skimmed milk powder prepared in Tris-buffered saline containing 0.05% Tween-20 (TBST) for 2 h at room temperature, the membranes were incubated with primary antibodies overnight at 4 °C. The next day, the membranes were washed six times with TBST on a shaker for 5 min, then incubated with species-appropriate horseradish peroxidase-conjugated secondary antibodies at room temperature for 1 h. After incubation, the membranes were washed six times with TBST on a shaker for 5 min, and detected with enhanced chemiluminescence. The level of protein was analyzed using Image Lab software (National Institutes of Health, Bethesda, MD, USA). The density of each band was normalized to its corresponding loading control (β-actin). 

### 2.12. Immunofluorescence Assays

For the tissue samples, paraffin-embedded cerebral cortices were sectioned, followed by deparaffinization in xylene and rehydration in decreasing grades of ethanol. The sections were blocked in bovine serum albumin (BSA). For the cell samples, primary cortical neurons (2 × 10^5^ cells per well) were seeded onto sterile cover slips in 24-well plates. After pretreating the cells for 1 h with 100 μM Pur, the cells were treated with or without 10 μM Cd for another 12 h. After washing in ice-cold PBS, the cells were fixed on coverslips with 4% paraformaldehyde. The cells were permeabilized with 0.5% Triton X-100 and blocked with 5% BSA. All of the tissue and cell samples were respectively labeled with rabbit anti-AIF (1:400 dilution) antibodies overnight at 4 °C, stained with Cy3-labeled goat anti-rabbit immunoglobulin G (IgG) (H+L) for 1 h at room temperature. The nuclei were stained with DAPI to visualize the nuclear morphology. The cells were viewed under a fluorescence microscope (TCS SP8 STED, Leica, Germany) to analyze the nuclear translocation of AIF.

### 2.13. Statistical Analyses

The results are expressed as the mean ± standard deviation (SD) from at least three independent experiments. Significance was calculated by a one-way analysis of variance (ANOVA) using SPSS 26.0 software followed by a Tukey’s test. The results were to be considered statistically significant at a threshold of *p* < 0.05.

## 3. Results

### 3.1. Pur Alleviates Cd-Induced Neuronal Injury

**Figure 1:**
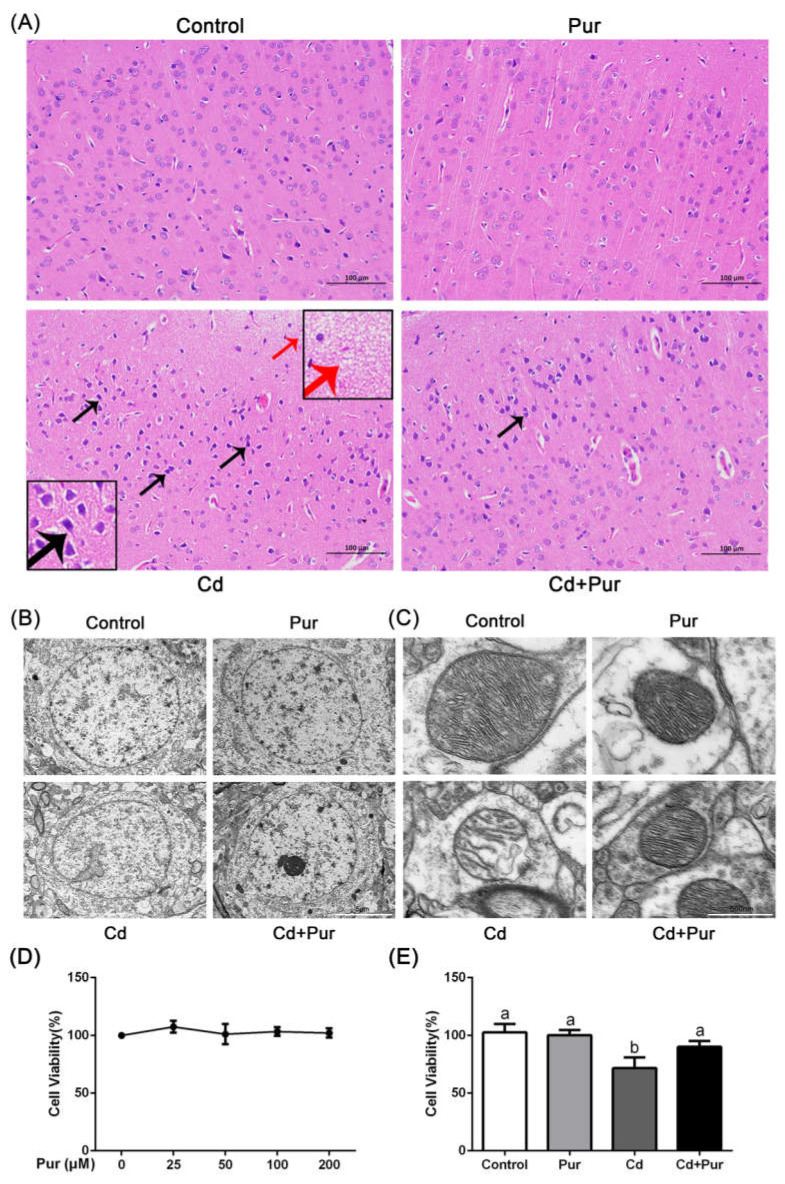
Pur alleviates Cd-induced neuronal injury. Rats were treated with Cd (50 mg/L) and/or Pur (200 mg/kg) for 90 days. (**A**) H&E staining of the rat cerebral cortices of each group. A period of 90 days of Cd exposure induced the formation of microscopic voids in the cortex (red arrows), neuronal deep staining, unclear boundaries between the cytoplasm and nuclei, as well as gaps around the neurons (black arrows). Treatment with Pur attenuated Cd-induced neuronal injury in the rat cerebral cortices. Scale bars: 100 μm. Representative electron microscopy images show the (**B**) nuclei and (**C**) mitochondria in the rat cerebral cortices of each group. Scale bars: (**B**) 5 μm and (**C**) 500 nm. (**D**) Primary cortical neurons were treated with different concentrations of Pur (0 μM–200 μM) for 12 h. Cell viability was detected using a CCK-8 assay. (**E**) Primary cortical neurons were pretreated with 100 μM Pur for 1 h, followed by exposure to 10 μM Cd for 12 h, after which the cell viability was detected via a CCK-8 assay. Data are expressed as the mean ± SD (*n* = 4). The means labeled without a common letter (a, b) are significantly different, *p* < 0.05.

Throughout the entire study, no abnormal behavior or symptoms were observed in any of the groups. Food intake and the body weight of the rats were monitored daily. Neither Cd nor Pur treatment had an obvious effect on food intake or weight gain in the rats (data not shown). To investigate neuroprotective effects of Pur, we evaluated the histological changes in the cerebral cortex by H&E staining (Figure 1A). We found that Cd caused microscopic voids in the cortex, neuronal deep staining, unclear boundaries between the cytoplasm and nuclei, as well as gaps surrounding the neurons. These findings confirm that the Cd-induced neurotoxicity model was successfully established. Moreover, Pur treatment decreased the Cd-induced microscopic voids in the cortex and neuronal deep staining. In addition, we examined the cortical neuron ultrastructure using transmission electron microscopy. As shown in Figure 1B,C, the nuclei were round, and both the complete mitochondrial structure and mitochondrial ridge were clearly visible in the control and Pur-alone samples. However, the Cd-treated samples exhibited obvious ultrastructural changes in the nucleus, nuclear indentation, and deformation. Furthermore, Cd also induced mitochondrial damage exhibited by distinct vacuolation and cristae disruption. It is important to note that Pur dramatically prevented Cd-induced morphological changes in the neurons, exhibiting a profile similar to that in the normal control. To further confirm the neuroprotective effects of Pur treatment, primary rat cerebral cortical neurons were isolated from fetal Sprague Dawley rats on day 18–19 of gestation. The cell viability was detected using a CCK-8 assay, and revealed that Pur alone had no significant effect on cell viability at any of the tested concentrations (Figure 1D). We selected 10 μM Cd and 100 μM Pur for subsequent experiments according to published reports and our previous experiments [9,21,25]. The results indicated that 100 μM Pur could significantly reduce Cd-induced cortical neuronal death (Figure 1E). Collectively, these results verified that Pur has a protective effect on Cd-induced neuronal injury both in vivo and in vitro.

### 3.2. Pur Reduces Cd Levels in the Rat Cerebral Cortex and Stimulates Cd Excretion

During this experiment, the average daily Cd consumption, calculated based on water intake, was similar between the Cd group and Pur and Cd co-treated group (Table 1). To assess the effect of Pur on Cd distribution, the Cd concentrations in the cerebral cortex, serum, urine, and feces of the rats were measured using an atomic absorption spectroscopy analysis. As shown in Table 1, exposure of the rats to Cd increased the levels of Cd in the cerebral cortex, serum, urine, and feces. Interestingly, co-administration of Pur significantly reduced the Cd levels in the cerebral cortex and serum, and increased the Cd levels in urine and feces of the rats exposed to Cd.

### 3.3. Pur Alleviates Cd-Induced Oxidative Stress in the Rat Serum and Cerebral Cortical Neurons

**Figure 2:**
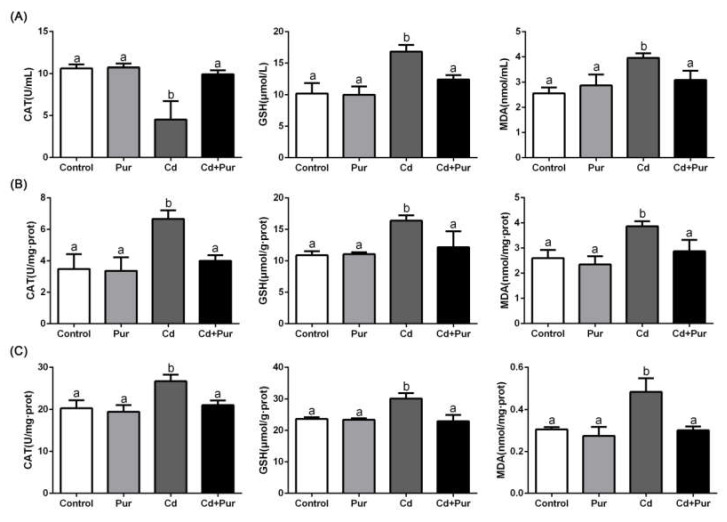
Pur alleviates Cd-induced oxidative stress in the rat serum and cerebral cortical neurons. The rats were treated with Cd (50 mg/L) and/or Pur (200 mg/kg) for 90 days. Primary cortical neurons were pretreated with 100 μM Pur for 1 h, followed by exposure to 10 μM Cd for 12 h. The levels of CAT activity, GSH, and MDA were evaluated in the rat serum (**A**), cerebral cortex (**B**), and primary cortical neurons (**C**). Data are expressed as the mean ± SD (*n* = 3). The means labeled without a common letter (a, b) are significantly different, *p* < 0.05.

Oxidative stress is considered to be a crucial mediator of Cd-induced neuronal injury. To explore whether Pur can inhibit Cd-induced oxidative stress in the entire body and the cerebral cortex in vivo, we assessed the parameters of oxidative stress in the serum and cerebral cortex in our rat model. As shown in Figure 2A, Cd exposure significantly decreased CAT activity and elevated the level of GSH and MDA in the serum. These levels were significantly alleviated by Pur administration. In contrast, compared with the control group, CAT activity and the level of GSH and MDA in the cerebral cortex were clearly increased following Cd exposure. This effect was alleviated by Pur administration (Figure 2B). In vitro experiments further confirmed that Pur significantly inhibited Cd-increased CAT activity and the level of GSH and MDA in the primary cortical neurons (Figure 2C). Taken together, Pur was able to significantly ameliorate Cd-induced impairment of the antioxidant status in the entire body and cerebral cortical neurons.

### 3.4. Pur Attenuates Cd-Induced Apoptosis via Inhibiting the Fas/FasL-Mediated Mitochondrial Apoptotic Pathway

**Figure 3:**
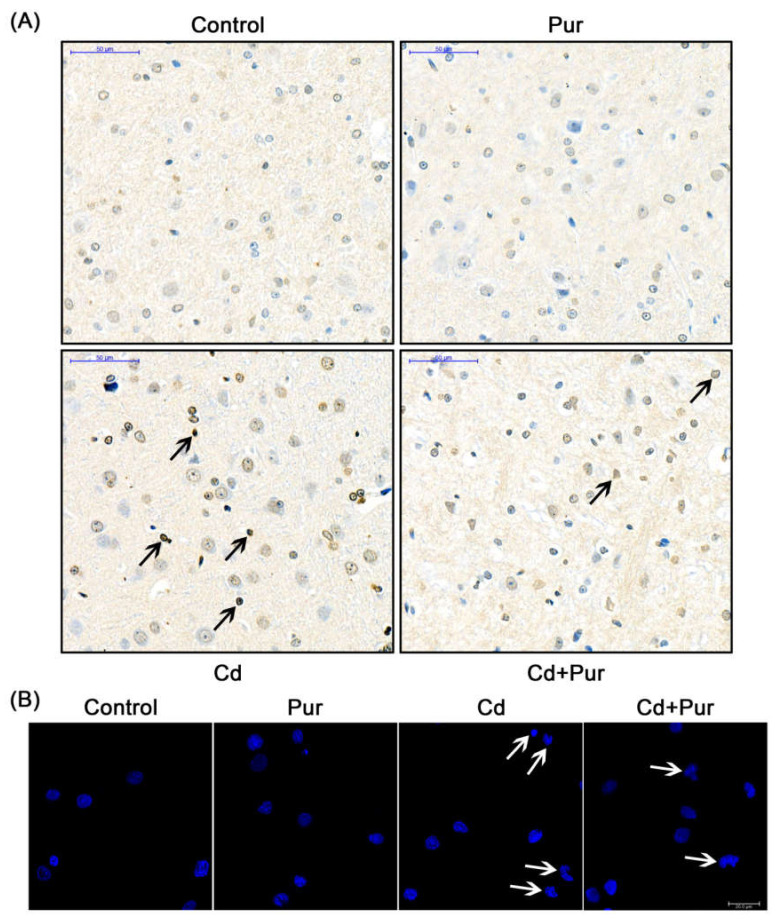
Pur attenuates Cd-induced apoptosis in rat cerebral cortical neurons. (**A**) Rats were treated with Cd (50 mg/L) and/or Pur (200 mg/kg) for 90 days. Apoptosis in the cerebral cortex was detected by a TUNEL assay. The TUNEL-positive cells were dark red (marked by black arrows). Scale bar: 50 μm. (**B**) Primary cortical neurons were pretreated with 100 μM Pur for 1 h, followed by 10 μM Cd exposure for 12 h. The morphology of the apoptotic cell nuclei was detected by DAPI staining (arrows point to apoptotic cells). Scale bars: 20 μm.

**Figure 4:**
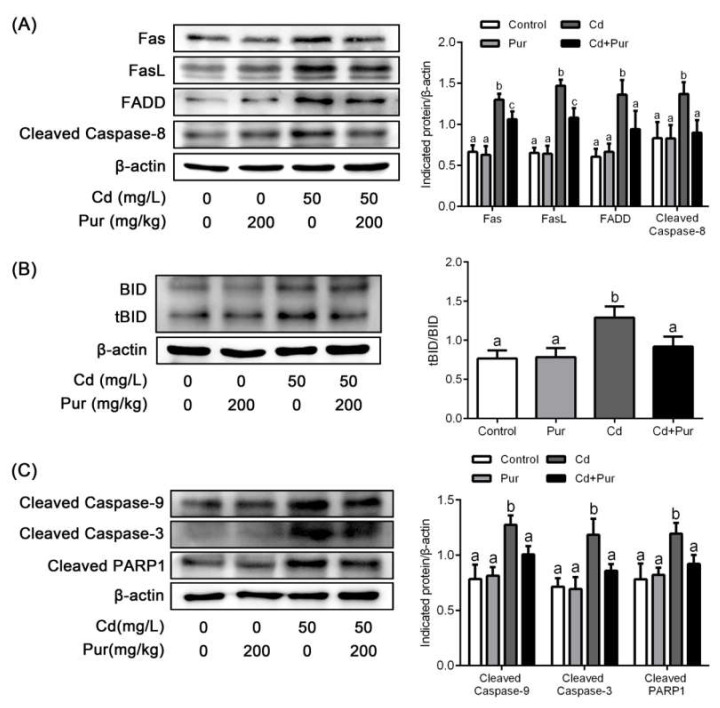
Pur attenuates Cd-induced apoptosis via inhibiting the Fas/FasL-mediated mitochondrial apoptotic pathway in the rat cerebral cortex in vivo. Rats were treated with Cd (50 mg/L) and/or Pur (200 mg/kg) for 90 days. (**A**) The levels of Fas, FasL, FADD, and Cleaved Caspase-8; (**B**) ratio of tBID/BID; and (**C**) the levels of Cleaved Caspase-9, Cleaved Caspase-3, and Cleaved PARP1 were detected by Western blot. Left panel: representative Western blot image; right panel, quantitative analysis (mean ± SD, *n* = 3). The means labeled without a common letter (a, b, c) are significantly different, *p* < 0.05.

**Figure 5:**
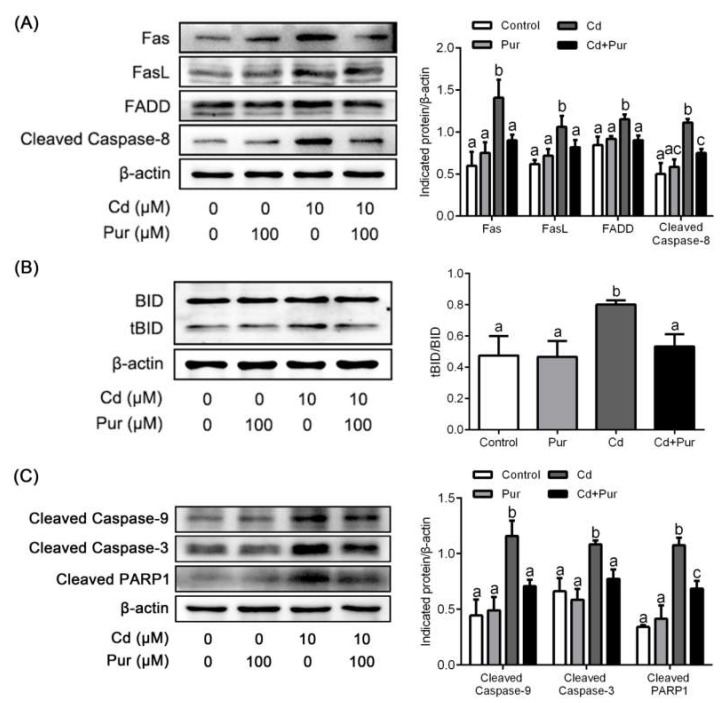
Pur attenuates Cd-induced apoptosis via inhibiting the Fas/FasL-mediated mitochondrial apoptotic pathway in primary cortical neurons in vitro. Primary cortical neurons were pretreated with 100 μM Pur for 1 h, followed by 10 μM Cd exposure for 12 h. (**A**) The levels of Fas, FasL, FADD, and Cleaved Caspase-8; (**B**) ratio of tBID/BID; and (**C**) the levels of Cleaved Caspase-9, Cleaved Caspase-3, and Cleaved PARP1 were detected by Western blot. Left panel: representative Western blot image; right panel: quantitative analysis (mean ± SD; *n* = 3). The means labeled without a common letter (a, b, c) are significantly different, *p* < 0.05.

**Figure 6:**
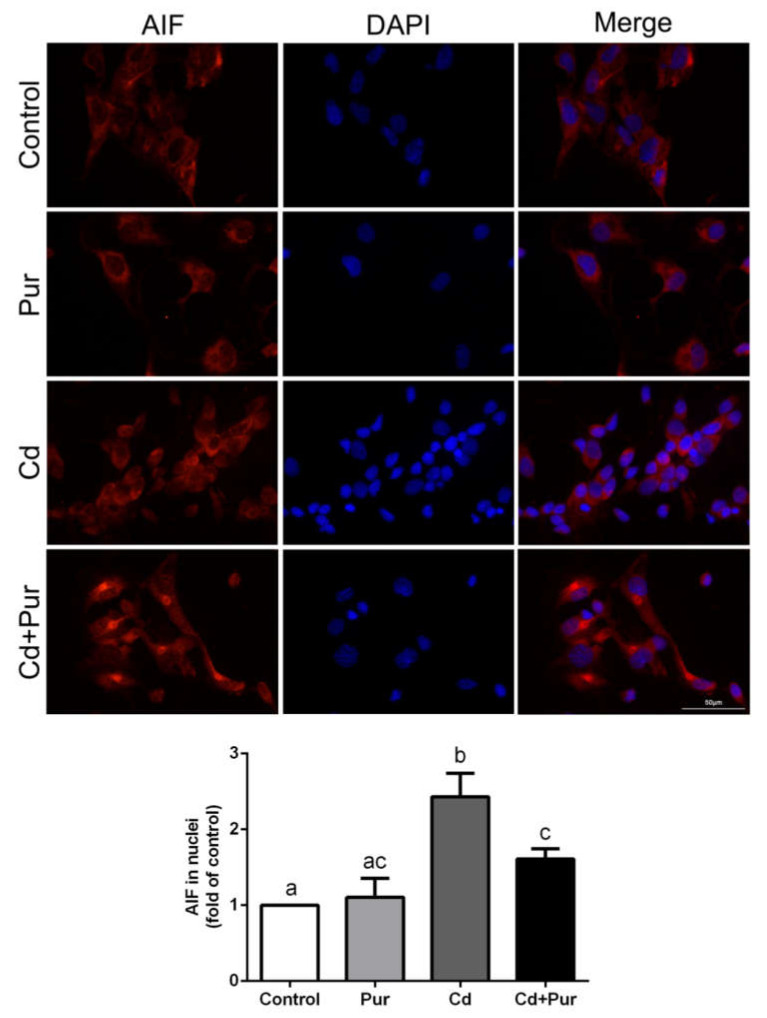
Pur prevents Cd-induced AIF nuclear translocation in the rat cerebral cortex in vivo. Rats were treated with Cd (50 mg/L) and/or Pur (200 mg/kg) for 90 days, after which the cerebral cortices were subjected to AIF immunofluorescence staining. Upper panel: representative immunofluorescence staining images; lower panel: quantitative analysis (mean ± SD; *n* = 3). The means labeled without a common letter (a, b, c) are significantly different, *p* < 0.05. Scale. bar: 50 μm.

**Figure 7:**
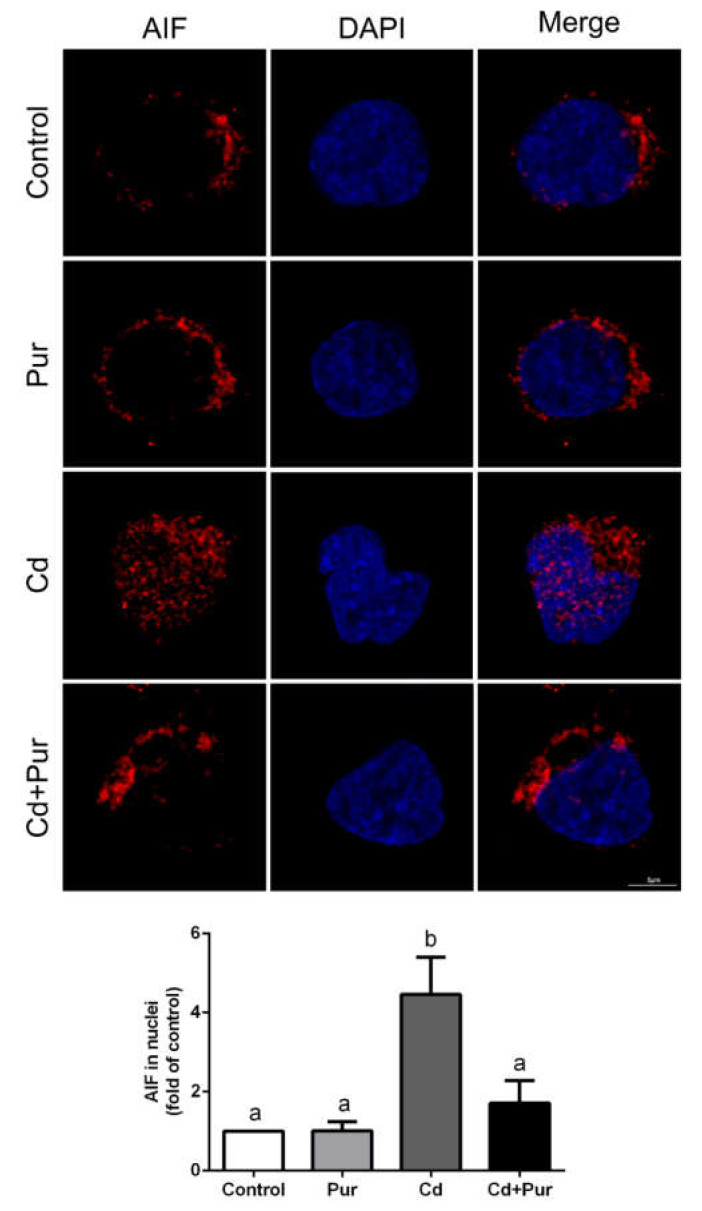
Pur prevents Cd-induced AIF nuclear translocation in primary cortical neurons in vitro. Primary cortical neurons were pretreated with 100 μM Pur for 1 h, followed by exposure to 10 μM Cd for 12 h. The neurons were subjected to AIF immunofluorescence staining. Upper panel: representative immunofluorescence staining images; lower panel: quantitative analysis (mean ± SD; *n* = 3). The means labeled without a common letter (a, b) are significantly different, *p* < 0.05. Scale. Scale bar: 5 μm.

Apoptosis is closely related to Cd-induced oxidative stress. To evaluate the effects of Pur on Cd-induced neuronal apoptosis, rats were treated with Cd (50 mg/L) and/or Pur (200 mg/kg) for 90 days. TUNEL staining revealed that Cd elevated the number of TUNEL-positive cerebral neurons, which was markedly attenuated following Pur treatment (Figure 3A). To further confirm the antiapoptotic properties of Pur, primary cortical neurons were pre-incubated with Pur (100 μM) for 1 h and subsequently treated with Cd (10 μM) for 12 h. The morphological analysis by DAPI staining revealed that Pur partially abolished Cd-induced typical apoptotic nuclear morphological changes; the presence of pyknotic nuclei or crescent shape appearance (Figure 3B). To gain greater insight into the neuroprotective effects of Pur mediated by reversing Cd-induced neuronal apoptosis, the level of proteins in the Fas/FasL and mitochondrial pathways were measured. The Western blot results (Figure 4A) demonstrated that Cd exposure caused a significant increase in the level of the pro-apoptotic proteins, Fas, FasL, FADD, and Cleaved Caspase-8 in the cerebral cortex of the rats, which was significantly attenuated by the co-administration of Pur. BID bridges the activation of cell surface death receptors and mitochondria. As shown in Figure 4B,C, the Cd-induced increase in the ratio of tBID/BID, as well as the cleavage of Caspase-9/3 and PARP1, were significantly inhibited by Pur treatment. In agreement with the in vivo results, Cd-induced upregulation of Fas, FasL, FADD, and Cleaved Caspase-8 were significantly attenuated by Pur in the primary cerebral cortical neurons in vitro (Figure 5A). Furthermore, a Cd-induced increase in the ratio of tBID/BID, as well as the activation of Caspase-9/3 and PARP1, were strikingly inhibited by Pur in vitro (Figure 5B,C). Moreover, AIF is a caspase-independent mitochondrial protein. Immunofluorescence staining (Figure 6 and Figure 7) showed that AIF was primarily restricted to the nuclei, as indicated by the colocalization with DAPI staining after Cd exposure. The co-administration of Pur significantly attenuated the Cd-induced AIF translocation to the nuclei both in vivo and in vitro. 

## 4. Discussion

Cd is a nonbiodegradable heavy metal associated with considerable environmental and occupational concerns. Increasing evidence has shown that Cd plays a critical role in neurobiology [8,26]. Cd causes neurotoxicity through various different mechanisms, and our previous studies show that Cd can induce oxidative stress and activate apoptotic pathways via the Fas/FasL, mitochondrial, mitogen-activated protein kinase (MAPK), mammalian target of rapamycin (mTOR) signaling pathways, and endoplasmic reticulum stress in neuronal cells [6,9,27,28]. Thus, the inhibition of oxidative stress and apoptosis is extremely important for attenuating Cd-induced neuronal injury. Pur has been described to possess potent neuroprotective activities due to its antioxidant and antiapoptotic properties [29]. The present study sought to reveal the efficacy of Pur-mediated protection against Cd-induced rat neuronal injury both in vivo and in vitro. Our results provide the first evidence that Pur exerted its neuroprotective effects by stimulating Cd excretion, ameliorating Cd-induced oxidative stress and apoptosis in rat cerebral cortical neurons.

Based on data from others’ and our previous experiments, we chose a treatment protocol of 50 mg/L Cd in drinking water for 90 days, with co-administration of 200 mg/kg·bw Pur by gavage [6,18]. During the experiment, the average intake of Cd was 3.7 mg/kg/day. The Cd dose administered was 4.2% of the LD_50_ for male rats, which was 88 mg/kg [30,31]. In addition, neither Cd or Pur treatment had any obvious effect on food intake and weight gain in the rats. To explore the mechanism of Pur-mediated protection, we evaluated the histological and ultrastructural changes of the cerebral cortices, and found that Pur alleviated the neuronal injury caused by Cd. Furthermore, we found that co-administration of Pur and Cd reduced the levels of Cd in the cerebral cortex of Cd-exposed rats. This effect may be attributed to the ability of Pur to protect the blood–brain barrier against dysfunction and prevent Cd from entering the cerebral cortex. Previous studies have revealed that antioxidants have the potential to protect the integrity of the blood–brain barrier [32,33]. The co-administration of Pur also reduced the Cd levels in the serum of Cd-exposed rats. Moreover, the urinary and fecal excretion of Cd were significantly increased in the Cd and Pur co-treated group compared with the Cd group. These findings indicate that Pur may be involved in stimulating Cd excretion, especially through feces. In agreement with studies involving cows and Wistar rats, most orally administered Cd is excreted via the feces [23,34]. This may be due to the fact that orally administered Cd is not fully absorbed, and Cd excretion through the feces does not involve complex body circulation. Om and Shim [23] reported that daidzein, an isoflavone that has phenolic rings in its structure, promoted Cd excretion from the body of rats. Therefore, as an isoflavone, Pur may have a high affinity to Cd and stimulate its excretion; however, further study is required to elucidate the molecular mechanism. In addition, our in vitro experiments also confirmed that Pur significantly reduced Cd-induced primary cortical neuronal death. Thus, additional studies are needed to explore the potential molecular mechanism by which Pur exerts its neuroprotective effects both in vivo and in vitro.

Multiple studies have reported that Cd is a strongly toxic metal capable of causing oxidative stress in the brain [35,36]. Since the brain is the most vulnerable organ to oxidative stress, such stress is an important factor in diseases of the nervous system [37,38]. There is an imbalance between high metabolic activity and low levels of endogenous antioxidants in the brain. The brain generates more ROS than any other tissue as it comprises only 2% of the total body weight in humans but consumes 20% of the body’s oxygen [39]. Extensive studies have shown that oxidative stress is strongly implicated in the pathogenesis of many neurological disorders, including Alzheimer’s disease and Parkinson’s disease [40,41]. Based on the anti-oxidative properties of Pur in nervous system diseases [42,43], we evaluated CAT activity, as well as the levels of GSH and MDA in the rat serum and cortical neurons. The results showed that Cd-induced lipid peroxidation and an impairment of the antioxidant defense system were rescued by Pur in vitro and in vivo. Notably, the elevated CAT activity and GSH level may be a self-defense response of the body against the Cd toxic effect. In contrast, Cd exposure significantly decreased CAT activity in serum. This diversity of results may be due to the fact that serum can inhibit Cd-induced CAT activation [44]. These results demonstrate that Cd-induced neuronal injury may be due to oxidative stress, which was alleviated by the antioxidative activity of Pur. 

As indicated above, Cd can induce oxidative stress in rat cerebral cortical neurons. Moreover, Cd-induced oxidative stress is closely related to the apoptosis in neuronal cells [45,46]. Hence, targeting apoptotic pathways may potentially reduce Cd-induced neuronal injury. The cell-death receptor (extrinsic) and mitochondrial (intrinsic) pathways are the two main signaling pathways that trigger apoptotic cell death. During apoptosis, the cell-death receptor pathway is activated by binding to cytokine ligands, such as FasL to Fas (CD95/APO-1), which activates Caspase-8 and as consequence, directly activates Caspase-3 or cleaves BID. BID is a BH3 domain-containing pro-apoptotic factor of the B-cell lymphoma 2 (Bcl-2) family, which bridges the activation of cell surface death receptors and mitochondria. Activated Caspase-8 in the Fas/FasL pathway cleaves BID, which induces the release of cytochrome c from mitochondria leading to subsequent damage [47]. The release of cytochrome c binds apoptotic protease activating factor-1 (Apaf-1), which forms a complex that activates Caspase-9 followed by Caspase-3 activation [48]. The activation of Caspase-3 cleaves PARP-1, which inhibits the repair of damaged DNA and results in cellular energy deprivation, ultimately promoting apoptotic cell death [49,50]. According to previous studies, Pur exerts neuroprotective effects through inhibiting Fas expression [51] or regulating the mitochondrial pathway [52] during cellular apoptosis. However, whether Pur has a neuroprotective effect against Cd-induced apoptosis has not been established. Moreover, during the exploration of the antiapoptotic properties of Pur in diseases of the nervous system, attention has rarely focused on the relationship between the Fas/FasL and mitochondrial pathways, especially regarding the critical role of BID. The present study demonstrates that Pur attenuates Cd-induced neuronal apoptosis both in vitro and in vivo. Cd significantly enhanced the level of Fas, FasL, FADD, and Cleaved Caspase-8, which was significantly reduced by Pur co-administration in vitro and in vivo. As expected, Cd upregulated the tBID/BID ratio and the level of Cleaved Caspase-9/3 and PARP1 proteins, whereas Pur significantly downregulated their levels both in vitro and in vivo. Although AIF is a flavoprotein normally confined to mitochondria, AIF is released into the cytosol and translocated into the nucleus after the induction of apoptosis, which mediates apoptotic functions through a caspase-independent pathway [53,54]. Pur has been shown to inhibit pancreatic β-cell apoptosis in a mouse model of T2D mellitus via the AIF-mediated caspase-independent apoptotic pathway [55]. Our results illustrate that Cd induced the nuclear translocation of AIF in cortical neurons, which were clearly inhibited by Pur both in vitro and in vivo. Taken together, we demonstrated that Cd induced neuronal apoptosis by activating the Fas/FasL-mediated mitochondrial apoptotic pathway in rat cerebral cortical neurons and Pur exerts neuroprotective inhibitory effects on these pathways both in vitro and in vivo. It should be highlighted that Pur is a phytoestrogen, and long-term estrogen use is associated with various complications (e.g., breast cancer, abnormal uterine bleeding, and adenocarcinoma of the endometrium) [56,57]. Therefore, the long-term use of Pur should be considered with caution.

## 5. Conclusions

**Figure 8:**
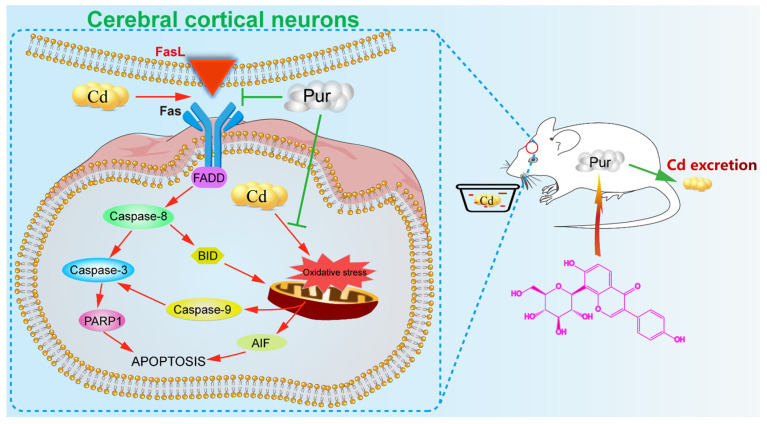
The mechanism by which Pur attenuates Cd-induced neuronal injury in rat cerebral cortical neurons.

The present study demonstrated for the first time that Pur significantly inhibited the Cd neurotoxicity by stimulating Cd excretion, ameliorating Cd-induced oxidative stress, and apoptosis in rat cerebral cortical neurons (Figure 8). Therefore, Pur may represent a promising novel neuroprotective agent.

## Figures and Tables

**Table 1:** Average daily Cd consumption and Cd concentrations in the cerebral cortex, serum, urine, and feces of the rats.

Groups	Control	Pur	Cd	Cd + Pur
Cd consumption (mg/kg body weight/day)	—	—	3.64 ± 0.52 ^a^	3.71 ± 0.48 ^a^
Cerebral cortical Cd (μg/g)	0.028 ± 0.008 ^a^	0.027 ± 0.007 ^a^	0.094 ± 0.014 ^b^	0.042 ± 0.005 ^a^
Serum Cd (μg/L)	0.35 ± 0.09 ^a^	0.32 ± 0.08 ^a^	1.52 ± 0.18 ^b^	0.87 ± 0.06 ^c^
Urinary Cd (μg/L)	1.59 ± 0.06 ^a^	1.66 ± 0.30 ^a^	36.46 ± 4.30 ^b^	63.96 ± 8.84 ^c^
Fecal Cd (μg/g)	0.56 ± 0.04 ^a^	0.62 ± 0.06 ^a^	148.34 ± 16.75 ^b^	219.96 ± 15.00 ^c^

Rats were treated with Cd (50 mg/L) and/or Pur (200 mg/kg) for 90 days. The average daily Cd consumption was calculated from the amount of water intake multiplied by the applied Cd concentration. The Cd concentrations in the cerebral cortex, serum, urine, and feces of the rats were measured using an atomic absorption spectroscopy analysis. Each value is presented as the mean ± SD (*n* = 3). The means labeled without a common letter (^a^, ^b^, ^c^) are significantly different, *p* < 0.05.

## Data Availability

The data presented in this study are available on request from the corresponding author Z.L.

## Abbreviations

AIF	apoptosis-inducing factor
BID	Bcl-2-interacting domain
CAT	catalase
CCK-8	cell counting kit-8
Cd	cadmium
CMC-Na	Carboxymethylcellulose sodium
DAPI	4′, 6-diamidino-2-phenylindole
FADD	Fas-associated death domain protein
FasL	Fas Ligand
GSH	glutathione
H&E	hematoxylin and eosin
MDA	malondialdehyde
PARP1	poly (ADP-ribose) polymerase-1
PBS	phosphate-buffered saline
Pur	puerarin
TBST	Tris-buffered saline containing 0.05% Tween-20
TUNEL	terminal deoxynucleotidyl transferase (TdT)-mediated deoxyuridine triphosphate (dUTP) nick-end labeling
